# Suprasellar Anterior-Posterior Diameter Optimizes the Use of Intraoperative MRI in Patients Undergoing Endoscopic Pituitary Surgery

**DOI:** 10.1227/ons.0000000000001319

**Published:** 2024-12-04

**Authors:** Cathal John Hannan, Christina Daousi, Mark Radon, Catherine E. Gilkes

**Affiliations:** * Department of Neurosurgery, The Walton Centre NHS Foundation Trust, Liverpool, UK;; ‡ Department of Endocrinology, Aintree University Hospital, Liverpool, UK;; § Department of Neuroradiology, The Walton Centre NHS Foundation Trust, Liverpool, UK

**Keywords:** Pituitary, PitNET, Intraoperative MRI, Endoscopic pituitary surgery

## Abstract

**BACKGROUND AND OBJECTIVES:**

Intraoperative MRI (iMRI) has been demonstrated to improve the extent of resection of pituitary neuroendocrine tumors resected using endoscopic endonasal approaches. We sought to establish if preoperative clinicoradiological parameters could be used to predict which patients are most likely to benefit from iMRI and thus allow more efficient use of this technology.

**METHODS:**

A prospectively maintained surgical database of all endoscopic pituitary tumor resections with iMRI guidance performed between May 2017 and September 2023 was accessed. Data were collected on clinical and radiological parameters that may predict reintervention after iMRI. Logistic regression models were constructed to assess the relationship between predictor variables and reintervention after iMRI.

**RESULTS:**

Seventy-three patients were included in the study. After review of the iMRI, 24/73 (33%) patients underwent surgical reintervention. The combined rate of gross total resection/near total resection was 64/73 (88%). The rate of biochemical cure of endocrine disease after surgery for a hormonally active tumor was 15/21 (71%). On univariate logistic regression analysis, the only factor significantly associated with reintervention after iMRI was the suprasellar anterior-posterior diameter (odds ratio 1.1, 95% CI 1.01-1.2, *P* = .030).

**CONCLUSION:**

Suprasellar anterior-posterior diameter ≥15 mm predicts the requirement for reintervention after endoscopic resection of pituitary neuroendocrine tumor. Use of this easily obtained radiological parameter will allow iMRI to be used in those patients who are most likely to benefit.

## Graphical abstract

**Figure:**
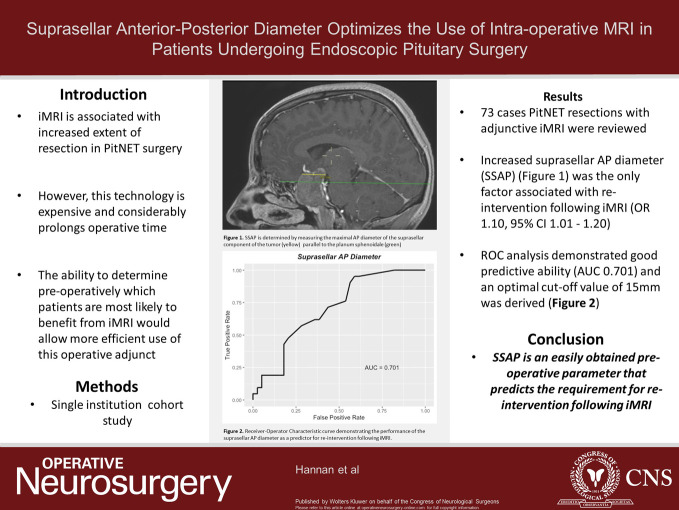

**ABBREVIATIONS:**
AP: anterior-posterior
EoR: extent of resection
GTR: gross total resection
iMRI: intraoperative MRI
NTR: near total resection
PitNETs: pituitary neuroendocrine tumors
SSAP: suprasellar AP.

The extent of resection (EoR) is a key determinant of outcome after surgery for pituitary neuroendocrine tumors (PitNETs).^1-3^ As pituitary surgery has evolved, a variety of surgical techniques and adjuncts have been used in an effort to improve resection rates; the use of endoscopic techniques, stereotactic image guidance, and medial cavernous sinus wall resection have all been demonstrated to improve outcomes, alongside increasing surgical experience.^4-7^

Intraoperative MRI (iMRI) has also been used with a view to improving EoR, and while a marked improvement in resection rates was observed in association with microscopic pituitary surgery, the potential additive benefit of iMRI when combined with endoscopy was initially regarded with skepticism.^8-10^ However, several recent publications have demonstrated that the use of iMRI increases the rate of gross total resection (GTR) of PitNETs, and even when a GTR is not possible, iMRI can facilitate a further resection to reduce tumor volume.^11-16^ There are undoubtedly shortcomings associated with the use of iMRI, including significant cost, prolonged operative time, and misinterpretation of blood products/hemostatic agents as residual tumor.^9,12,17,18^

In our institution, we have aimed to use iMRI in every endoscopic PitNET resection since May 2017. However, the use of this equipment is associated with significant resource allocation issues; this iMRI scanner is also used for general diagnostic purposes, and its use for patients under general anesthesia limits its capacity for other patients. Moreover, because of the prolonged operative time associated with the use of iMRI, generally only one pituitary tumor resection can take place on each operating list, which affects the duration of the surgical waiting list. In an effort to abrogate the resource allocation issues associated with the use of iMRI, we aimed to establish if any preoperative clinicoradiological factors could accurately predict the need to reintervene after iMRI.

## METHODS

### Patient Population

A prospectively maintained database of all endoscopic skull base cases performed by the senior author was accessed, and data pertaining to consecutive endoscopic pituitary tumor resections with adjunctive iMRI performed between May 2017 and September 2023 were extracted. Relevant institutional approvals were obtained, and as only fully anonymized data were extracted, the requirement for individual patient consent was waived.

### Surgical Technique and iMRI Protocol

As described previously, the head was fixed in an MRI-compatible head holder, which integrates with a specifically designed head coil (NORAS).^12^ Endoscopic resection of the tumor then proceeds in the standard fashion. Rarely, a staged approach was adopted, whereby an initial endoscopic endonasal resection was followed by a craniotomy for giant tumors with significant extrasellar extension.

The iMRI was performed when the operating surgeon determined that a maximal safe resection had been obtained and after the completion of skull base repair. The patient is then transferred to the 3 T iMRI scanner (Siemens) under general anesthesia, and the following sequences are obtained: T1 w Magnetization Prepared - RApid Gradient Echo 1.2 mm isotropic 3-dimensional (3D), T2w sampling perfection with application optimized contrasts using different flip angle evolution 0.9-mm isotropic 3D, Axial 3-mm segmented echo planar imaging diffusion-weighted imaging (b = 1000 s/mm^2^). If, after review of the initial imaging, the presence/absence of resectable residual tumor remains unclear, the following postcontrast sequences are obtained: T1w + C Magnetization Prepared - RApid Gradient Echo 1.2 mm isotropic 3D, T1w + C spectral attenuated inversion recovery sampling perfection with application optimized contrasts using different flip angle evolution (fat suppressed) 0.6-mm isotropic 3D (sella).^12^

If, after review of the imaging by a consultant neuroradiologist, resectable residual tumor was felt to be present, the patient was transferred from the iMRI scanner back to the operating theater and further surgical resection was performed. If the MRI scan was adjudged not to demonstrate resectable residual, the patient was awoken from anesthesia. In cases where surgical reintervention was prompted by iMRI, all resected tissues were sent separately for histopathological analysis to determine if residual tumor was present.

### Follow-up

Typically, the first postoperative MRI scan (T1w + C 2 mm coronal and sagittal) was obtained 3 months postoperatively. Patients then received radiological and endocrinological follow-up as appropriate, with surveillance imaging performed annually. The duration of clinical follow-up was defined as the time from surgery until last outpatient review. The duration of radiological follow-up was defined as the time from surgery until most recent surveillance imaging.

### Data Collection

**FIGURE 1.**
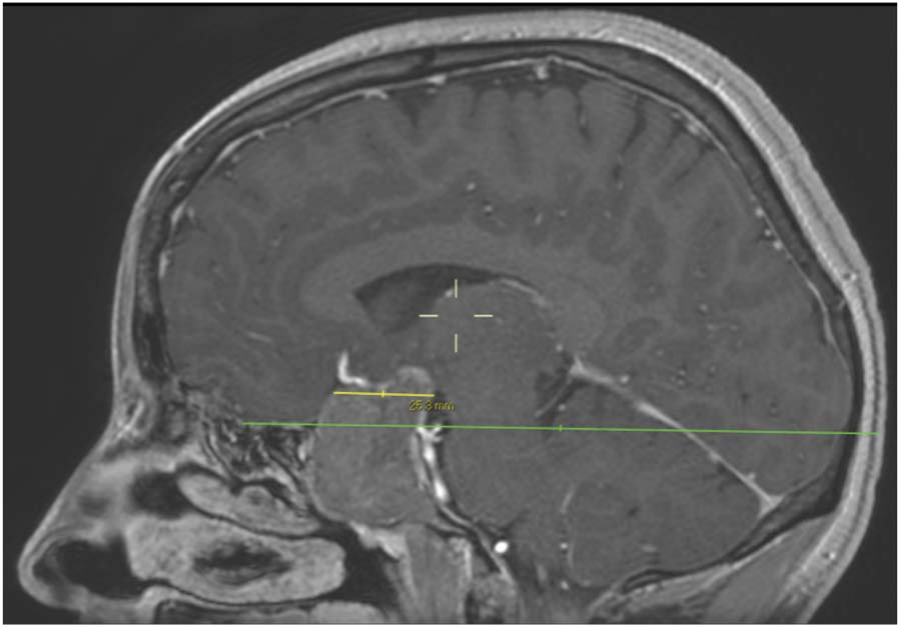
Contrast-enhanced sagittal T1-weighted MRI demonstrating a pituitary macroadenoma with suprasellar extension. The suprasellar anterior-posterior diameter (yellow line) is determined by measuring the longest anterior-posterior distance above and parallel to the planum line (green line).

Preoperative tumor volumes were obtained from thin slice (1-1.2 mm slice width) T1-weighted MRI with contrast using the Brainlab Elements (Brainlab) software to manually contour the tumors. Tumoral maximal diameter was calculated by measuring the maximum diameter in any plane of preoperative imaging. The modified Knosp grade of cavernous sinus invasion was calculated.^19^ The suprasellar anterior-posterior (SSAP) diameter was calculated by measuring the maximal anterior-posterior distance above and parallel to a line extending along the planum sphenoidale (Figure 1).^20^

In the absence of an established core outcome set for pituitary surgery, tumor resections were classified as GTR when no tumor residuum was evident on iMRI/postoperative MRI, near total resection (NTR) when a small volume of tumor residuum with maximal diameter ≤3 mm was present, and subtotal resection when the maximal diameter of tumor residuum exceeded 3 mm.^21^

Operative time was defined as the time between patient transfer from the anesthetic room into the operating theater and the end of the case, including the time taken for all iMRI safety checklists, transfer to/from the scanner, and scanning time.

For the patients in the study with acromegaly, remission was defined as normalization of insulin-like growth factor 1 to within the age-adjusted normal range and nadir growth hormone level <0.4 mcg/L on oral glucose tolerance testing performed ≥3 months postsurgery.^22^ For patients with Cushing's disease, morning serum cortisol levels were measured on 2 separate occasions within 7 days of tumor resection. Levels <50 nmol/L are considered highly likely to be associated with remission, but these initial results always correlate with 24-hour urine-free cortisol excretion, and 1-mg overnight dexamethasone suppression test results in an outpatient setting. Periodic clinical and biochemical evaluation is performed for all patients to help ascertain persistence of hypocortisolism, eucortisolism, or hypercortisolism in the postoperative period.^23^

### Statistical Analysis

Linear logistic regression models were constructed to examine the relationship between predictor variables and reintervention after iMRI. Basic spline logistic regression techniques were then used to optimize model fit. Receiver operating characteristic curves were devised to assess the predictive performance of identified predictor variables when compared with random assignment. The optimal cutoff value for continuous predictor variables was determined by calculating Youden's J statistic for a range of values, with the value producing the highest J statistic selected. All statistical analyses were performed in R v.4.3.2 (The R Foundation for Statistical Computing). All work was performed in accordance with the Strengthening the Reporting of Observational Studies in Epidemiology recommendations.^24^

## RESULTS

Between May 2017 and September 2023, 143 endoscopic endonasal resections of pituitary adenomas were undertaken by the senior author, with the aspiration to use iMRI for each case. However, the iMRI was not used in circumstances where the iMRI was not available (equipment malfunction or being used by another surgeon) or where MRI was not suitable because of patient factors (eg, morbid obesity). Use of the iMRI was also restricted during the COVID-19 pandemic.

Ultimately, 73 patients underwent iMRI-assisted endoscopic resection of a pituitary tumor in our institution during the study period. Baseline demographic and clinical details of this cohort are displayed in Table 1. Most of the patients underwent resection of a macroadenoma (56/73, 78%), with giant adenomas (11/73, 15%) and functioning microadenomas (5/73, 7%) making up the remainder of the cohort. Overall, 26/73 (36%) of the cohort had hormonally active tumors, and most of those were diagnosed with acromegaly (19/73, 26%), whereas a much smaller proportion was diagnosed with Cushing's disease (3/73, 4%) or other secretory tumors (4/6, 6%), eg, Thyroid Stimulating Hormone-PitNET.

**TABLE 1.** Baseline Characteristics of the Study Cohort

Characteristic	Total (n = 73)		
Female (%)	33 (45)		
Median age (IQR)	50 (21)		
Microadenoma (%)	5 (7)		
Macroadenoma (%)	57 (78)		
Giant adenoma (%)	11 (15)		
Median tumor diameter/mm (IQR)	25 (12)		
Median tumor volume/cm^3^ (IQR)	6.3 (4.7)		
Hormonally active (%)			
Acromegaly (%)	26 (36)		19 (26)
Cushing's (%)			3 (4)
Other (%)			4 (6)
Knosp grade			
0 (%)	6 (8)		
1 (%)	4 (6)		
2 (%)	23 (32)		
3a (%)	22 (30)		
3b (%)	10 (14)		
4 (%)	8 (11)		
Suprasellar/clival extension (%)			
Suprasellar (%)	61 (84)	59 (81)	
Clival (%)		13 (18)	
Suprasellar and clival (%)		11 (15)	
Median suprasellar anterior-posterior diameter/mm (IQR)	18 (10)		
Previous pituitary surgery (%)	2 (3)		
Median duration of radiological F/U/mo (IQR)	33 (40)		
Median duration of clinical F/U (IQR)	41 (34)		

**FIGURE 2.**
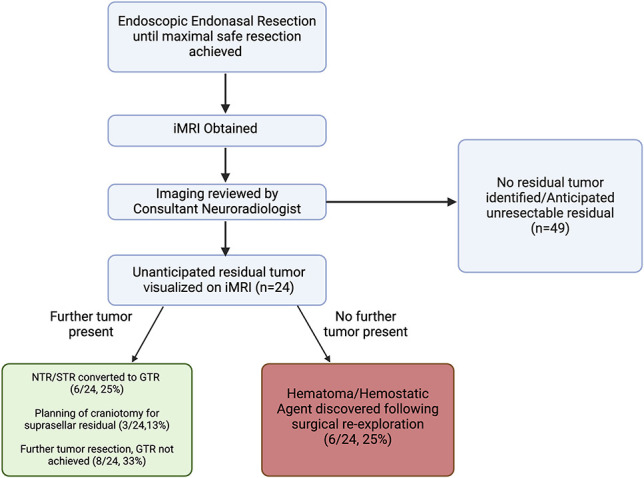
Flow diagram demonstrating the surgical and radiological workflow. After the operating surgeon feels that a maximal safe resection has been achieved, an iMRI is obtained and reviewed by a consultant radiologist. In this series, 24 iMRIs were felt to demonstrate resectable residual tumor. Further tumor was resected in 18/24 cases, but in 6/24 cases, the abnormalities on iMRI were found to be hematoma or hemostatic agents. GTR, gross total resection; iMRI, intraoperative MRI; NTR, near total resection; STR, subtotal resection.

After review of the iMRI, 24/73 (33%) patients were found to have an unanticipated tumor residuum, which prompted a return to the operating room. Further tumor was resected in 18/24 (75%) of these cases. The use of iMRI guided the conversion of a NTR/STR to a GTR in 6/24 (25%) cases and facilitated the planning of a craniotomy for a large suprasellar residual in 3/24 (13%) cases. In a further 8/24 (33%) cases, iMRI demonstrated residual tumor that was resected, although not to the extent that a GTR was achieved. In 6/24 (25%) cases, further exploration of the operative site did not reveal any residual tumor and the area of suspected tumor on MRI was found to be composed of hematoma/hemostatic agents (Figure 2).

Overall, the rate of GTR/NTR was 64/73 (88%). A total of 10/73 (14%) patients have required reintervention in the postoperative period, most of whom have undergone fractionated radiotherapy for residual/recurrent tumors. The overall rate of recurrence or regrowth of residual tumor was 2/73 (3%) during a median radiological follow-up period of 33 months (IQR 39). The rate of biochemical cure of endocrine disease after surgery for a hormonally active tumor was 15/21 (71%) (5 patients are currently undergoing postoperative endocrinological testing to establish if remission has been achieved). During a median clinical follow-up period of 41 months (IQR 34), there have been no instances of biochemical recurrence.

**FIGURE 3.**
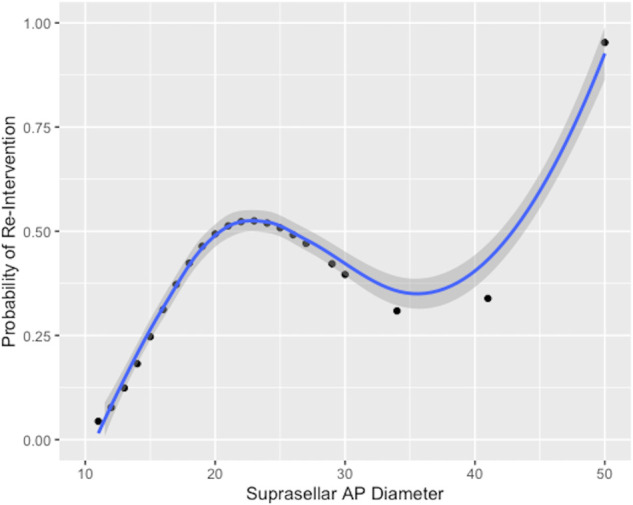
Graphical representation of the basic spline logistic regression model, demonstrating the variation in the probability of reintervention after intraoperative MRI with increasing suprasellar AP diameter. Gray shading indicates 95% CI of the estimate. AP, anterior-posterior.

**FIGURE 4.**
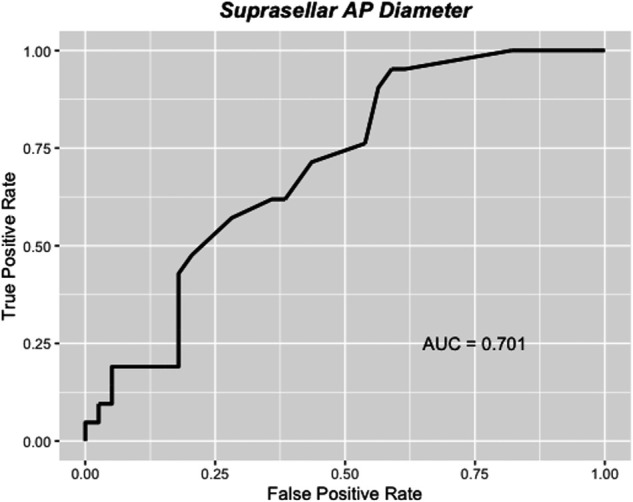
Receiver-operator characteristic curve demonstrating the performance of the suprasellar AP diameter as a predictor for reintervention after intraoperative MRI. The area under the curve was calculated to be 0.701, and the suprasellar AP diameter was found to significantly outperform a random classifier (*P* = .008). AUC, area under the curve; AP, anterior-posterior.

**FIGURE 5.**
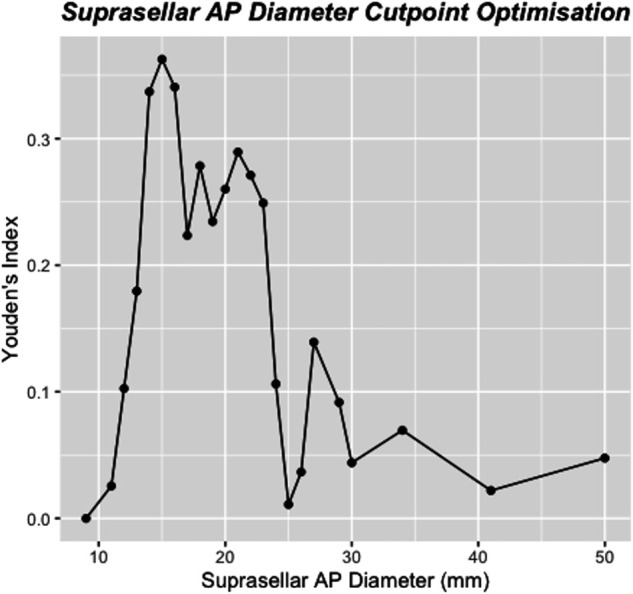
Line graph demonstrating the performance of potential cut points in suprasellar AP diameter. A cut point of 15 mm was found to be optimal as assessed by Youden's J statistic. AP, anterior-posterior.

On univariable logistic regression analysis, the only factor significantly associated with reintervention after iMRI was the SSAP diameter (odds ratio 1.1, 95% CI 1.01-1.2, *P* = .030) (Table 2). The probability of reintervention after iMRI was found to increase linearly until the SSAP diameter reached 23 mm, after which the small number of cases might have affected the precision of the estimate (Figure 3). Receiver operating characteristic analysis of the SSAP as a predictor of reintervention after iMRI produced an area under the curve of 0.701 (*P* = .008), indicative of a parameter with good discriminative ability^25^ (Figure 4). By calculating the value of Youden's J statistic, multiple cutoff values of SSAP were trialed and a value of ≥15 mm was determined to be optimal (Figure 5). This cutoff point was found to have a sensitivity of 95% and a specificity of 41% in predicting surgical reintervention after iMRI, and use of this cutoff value in the current series would have reduced the use of iMRI by 27% while missing only one case where reintervention was required after iMRI.

**TABLE 2.** Predictors of Reintervention After Intraoperative MRI

Parameter	Univariate logistic regression		
	Odds ratio	95% CI	Sig.
Sex (female vs male)	1.45	0.54-3.9	0.460
Age	0.99	0.96-1.03	0.666
Tumor size classification			
Microadenoma	Reference		
Macroadenoma	0.89	0.1-7.5	0.889
Giant adenoma	1.31	0.34-5.08	0.695
Tumor max diameter (mm)	1.04	0.99-1.09	0.095
Tumor volume (cm^3^)	1.03	0.98-1.07	0.207
Suprasellar extension	0.585	0.14-2.46	0.452
Clival extension	1.38	0.39-4.91	0.624
Nodular extension	2.22	0.72-6.86	0.164
Suprasellar height	1.13	0.84-1.09	0.534
Suprasellar anterior-posterior diameter	**1.10**	**1.01-1.20**	**0.030**
T2SIR	0.32	0.00-5.80	0.194
Knosp 3A-4	2.04	0.73-5.69	0.175
Hormonally active	0.69	0.24-1.99	0.493
Previous pituitary surgery	2.18	0.13-36.51	0.587

Bold indicates statistically significant comparison

## DISCUSSION

### Main Findings

In the first comprehensive analysis of factors associated with reintervention after iMRI during endoscopic pituitary surgery, we have demonstrated that the SSAP is strongly associated with the requirement for further surgical reintervention after iMRI (Figures 3 and 4). Moreover, we have identified an optimal cutoff value of 15 mm for SSAP, which is associated with a sensitivity of 91% and a specificity of 41% in the prediction of reintervention after iMRI (Figure 5). Had it have been applied to this cohort, it would have reduced the burden of the performed iMRI by 27%, which would have permitted the MRI scanner to have been used for other applications and facilitated more efficient utilization of odds ratio time. In our view, this easily obtainable radiological measure will be useful clinically in determining which patients stand to benefit the most from the use of iMRI as an adjunct and allow more efficient utilization of limited healthcare resources. On the basis of our results, we recommend the use of iMRI as an adjunct for the endoscopic resection of pituitary tumors with a SSAP of ≥15 mm and giant adenomas that may require a staged resection including a craniotomy to ensure complete tumor resection and all hormonally active tumors (assuming that they were visible on the preoperative MRI).

### Results in the Context of the Literature

**FIGURE 6.**
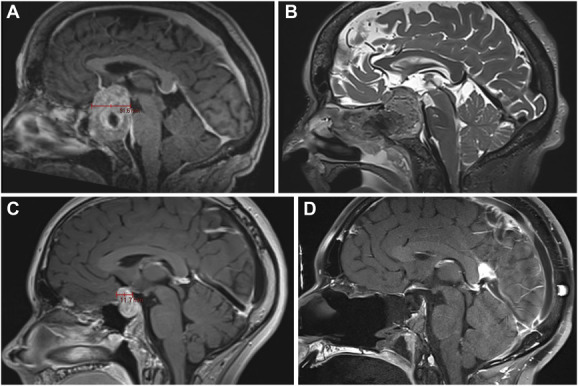
**A**, Contrast-enhanced sagittal T1-weighted preoperative MRI demonstrating a large PitNET with a suprasellar AP diameter of 31.5 mm. **B**, T2-weighted intraoperative MRI demonstrating central debulking of the tumor, with a significant suprasellar residual component requiring operative reintervention. **C**, Contrast-enhanced sagittal T1-weighted preoperative MRI demonstrating a PitNET with suprasellar extension and a suprasellar AP diameter of 11.7 mm. **D**, T1-weighted intraoperative MRI demonstrating gross total resection. AP, anterior-posterior; PitNET, pituitary neuroendocrine tumor.

The SSAP was first reported as a predictive factor for the resection of large and giant PitNETs by Peto et al,^26^ who identified a significantly larger SSAP in tumors that underwent STR when compared with those that were completely resected. In a further publication from the same group, Park et al^20^ expanded on these findings and demonstrated that a higher SSAP was associated with a decreased rate of GTR when applied to all pituitary tumors with suprasellar extension. The authors identified a cutoff value for SSAP of 23.7 mm as being optimal in predicting if a GTR could be achieved and posited that in cases with increased SSAP, “blind spots” at the anterior-superior and posterior-superior margins of the tumor would emerge because of descending folds of arachnoid obscuring the surgical field as the more accessible aspects of the tumor were debulked. Figure 6 illustrates this point, demonstrating a large suprasellar residual after debulking of a large PitNET with significant suprasellar extension requiring operative reintervention after iMRI.

The significance of the anterior-posterior diameter in predicting the EoR in PitNETs has also been recognized by others; the Hardy, Age, Clival, Knosp, Depth score is a recently developed, validated scoring system for the prediction of GTR after endoscopic pituitary surgery. On the basis of their multicenter analysis of 277 patients, the authors were able to demonstrate that increasing tumor depth (AP diameter) was significantly associated with a decreased likelihood of obtaining a GTR.^27^ Similarly, a further scoring system derived from the multicenter TRANSSPHER study data set determined that nodular extension of the PitNET into the suprasellar space was independently negatively predictive of GTR although we did not demonstrate a relationship between nodular extension and the requirement for reintervention after iMRI in our data set (Table 2).^28^ With specific respect to nodular extension, it may be the case that cases with nodular extension are more likely to be associated with small volume residuals located in anatomically inaccessible areas; while this may be identified on iMRI, the operating surgeon may feel that reintervention for small residuals in areas not easily reached using an endoscopic approach are not warranted.

The only other study that has attempted to optimize patient selection and guide the use of iMRI in patients undergoing endoscopic resection of PitNETs identified the Zurich pituitary score (ZPS) as being predictive of conversion to GTR after iMRI.^14^ The ZPS is calculated by determining the ratio of the maximum horizontal diameter of the tumor to the intercarotid distance at the horizontal segment of the cavernous carotid artery, with a higher ratio indicating a narrower surgical corridor and increased inaccessibility of the tumor.^29^ In seeking to validate the use of the ZPS to guide patient selection for iMRI, the authors noted that those patients with a lower ZPS grade were more likely to have their resection converted to a GTR after iMRI.^14^ However, the ZPS was the only predictive factor assessed in this study and we believe that our analysis of multiple predictive factors offers a more comprehensive assessment of this clinical question.

Historically, it was recommended that postoperative MRI should be delayed for at least 3 months after resection of a PitNET, in view of the difficulty in interpreting early postoperative studies because of the presence of hematoma, fat packing, and postoperative changes.^30^ However, these recommendations were based on data obtained before the widespread adoption of more advanced magnetic resonance techniques such as fat saturation imaging, which facilitate distinction between residual adenoma and postoperative changes.^31,32^ More recent studies have demonstrated the value of early postoperative MRI in the detection of residual adenoma requiring reoperation, which can be performed during the same admission before the development of postoperative adhesions/scar tissue.^33-35^ In our view, iMRI extends this advantage further, by permitting the further tumor resection during the same anesthetic. Although this undoubtedly prolongs operative times, in appropriately selected cases, the use of iMRI is invaluable in allowing ultra-early detection of residual tumor and addressing this immediately.^12^

### Limitations

This study is subject to a number of limitations. First, it is based on the experience of a single surgeon and is therefore subject to the inherent limitations of such studies; the findings outlined in this article should be subjected to robust external validation. Specifically, our calculation of the proportion of cases where iMRI could have been avoided, had our 15-mm SSAP cutoff value been applied, is vulnerable to bias as it was applied to the same cohort of patients from which our predictive model was derived, and this finding has not been externally validated. Second, although our proposed SSAP cutoff value of 15 mm has a very high sensitivity of 95%, it may be criticized for a relatively low specificity of 41%. However, in this particular clinical setting where a test with a very low rate of false negatives is desirable, sensitivity is justifiably weighted more heavily than specificity.^36^ Although a false-positive result on iMRI may prompt an unnecessary surgical reintervention, data from a previous publication on this topic have demonstrated no increase in postoperative complications in association with surgical reintervention after iMRI.^12^ We have adapted our imaging protocol to help improve the specificity of the intraoperative imaging: diffusion weighted imaging can be used to assess for cellularity, which helps to distinguish cellular tissue from hematoma or acellular material such as gelatin; the use of a very high-resolution fat-suppressed sequence is also useful for distinction of fat and devascularized tissue from viable tissue.^37,38^

## CONCLUSION

SSAP is an easily obtained preoperative parameter that predicts the requirement for reintervention after iMRI in the setting of endoscopic pituitary surgery. Use of this measurement may allow the direction of iMRI toward those patients who are most likely to benefit from its use and facilitate the more efficient allocation of limited clinical resources.
